# Development of Antioxidant and Antihypertensive Properties during Growth of *Lactobacillus helveticus*, *Lactobacillus rhamnosus* and *Lactobacillus reuteri* on Cow’s Milk: Fermentation and Peptidomics Study

**DOI:** 10.3390/foods10010017

**Published:** 2020-12-23

**Authors:** Anna V. Begunova, Olga S. Savinova, Olga A. Glazunova, Konstantin V. Moiseenko, Irina V. Rozhkova, Tatyana V. Fedorova

**Affiliations:** 1 Federal State Budgetary Scientific Institution “All-Russian Research Institute of Dairy Industry”, 115093 Moscow, Russia; abegunova@yandex.ru (A.V.B.); irina.v.rozhkova@gmail.com (I.V.R.); 2 A.N. Bach Institute of Biochemistry, Research Center of Biotechnology of the Russian Academy of Sciences, 119071 Moscow, Russia; savinova_os@rambler.ru (O.S.S.); olga.a.glas@gmail.com (O.A.G.); fedorova_tv@mail.ru (T.V.F.)

**Keywords:** *Lactobacillus*, milk fermentation, proteolytic activity, antioxidant activity, angiotensin-converting enzyme inhibitory activity (ACE-I), peptidomics, bioactive peptides

## Abstract

Bioactive peptides derived from milk proteins are an active research area. Exhibiting numerous positive physiological effects on digestive, cardiovascular, immune and nervous systems, these peptides thought to be one of the most promising ingredients for functional food. Generally, these peptides are inactive within the parent proteins and can be liberated during milk fermentation by the specific proteolytic systems of various *Lactobacillus* spp. Here we present the study of milk fermentation by *Lactobacillus helveticus* NK1, *Lactobacillus rhamnosus* F and *Lactobacillus reuteri* LR1 strains. It was demonstrated that the antioxidant activity of the milk fermented by these strains concomitantly increased with the strains’ proteolytic activity. For the angiotensin I-converting enzyme (ACE) inhibitory activity, the same tendency was not observed. Although the proteolytic activity of *L. helveticus* NK1 was two times higher than that of *L. rhamnosus* F, the milk fermented by these strains showed comparable ACE inhibition. The analysis of the peptide profiles of the fermented milk samples allowed us to hypothesize that some previously unreported peptides can be produced by *L. rhamnosus* F. In addition, it was demonstrated that these potential ACE-inhibiting peptides originated from the C-terminus of α_S2_-casein.

## 1. Introduction

Nutraceuticals are any compounds derived from food sources and possessing extra health benefits in addition to their basic nutritional value [1]. Typically, these compounds are present by non-specific biological agents used to promote general well-being, control symptoms and prevent malignant processes [2]. In this regards, nutraceuticals can be generally considered as an alternative to pharmaceuticals (specifically designed and usually chemically synthesized health-promoting and symptom-alleviating substances) [3].

Over the past decade, interest has risen in fermented dairy foods as a potent source of nutraceuticals [4]. The nutraceutical values of these foods had been mainly attributed to bioactive peptides encrypted within dairy proteins [5]. During fermentation these peptides are released by the specific sets of proteases—cell envelope proteinases, CEPs—possessed by lactic acid bacteria (LABs) [6]. Currently, the list of the proven health-promoting properties of dairy peptides includes, but is not limited to, antioxidant, antihypertensive, antimicrobial, antithrombotic and immunomodulatory properties [7].

Traditionally, the starter cultures for dairy fermentation are selected based only on the rheological, organoleptic and nutritional characteristics of the final product. However, the potential of LABs to produce bioactive peptides forced the reconsideration of these requirements. Nowadays, many scientists all over the world are involved in the search for new strains of LABs that can be either added to the traditional starters for their enrichment in bioactive peptides or used alone to produce bioactive premixes [8,9,10].

In our previous work, we demonstrated that *Lactobacillus helveticus* NK1, *Lactobacillus rhamnosus* F and *Lactobacillus reuteri* LR1 strains exhibit good safety and technological performance, as well as provide good probiotic properties to the final products [11,12]. In this article, the proteolytic activity of these LABs and their ability to release bioactive peptides with antioxidant and angiotensin I-converting enzyme inhibitory (ACE-I) properties were investigated.

## 2. Materials and Methods

### 2.1. Preparation of Fermented Milk Samples

*L. helveticus* NK1, *L. rhamnosus* F, and *L. reuteri* LR1 strains were obtained from the Microorganism Collection of the All-Russia Research Institute of the Dairy Industry (VNIMI, Moscow, Russia). The sequences of the 16S ribosomal RNA genes of these strains can be found at the GeneBank accessory numbers MT448799, MN994629 and MN994628 for *L. helveticus* NK1, *L. rhamnosus* F, and *L. reuteri* LR1, respectively.

For all fermentations, one liter of a reconstituted skim milk (RSM) was prepared by adding the appropriate amount of skim milk powder to water and heating at 85 °C for 30 min with stirring. The heat-treated milk was then cooled to approximately 40 °C. The sterile RSM was aseptically inoculated with 1% (*v/v*) of each strain (approximately 10^7^ cells·mL^−1^) and incubated at 37 °C for 72 h. Samples were taken for analysis at 0, 6, 16, 24, 48, and 72 h of incubation and the change in pH was measured at each time point.

The cell population of each LAB was determined by counting the colony-forming units (CFU) on MRS agar after incubation at 37 °C for 48–72 h under anaerobic conditions using GasPak EZ anaerobe container systems (Becton, Dickinson and Company, Sparks, MD, USA).

All fermentations were performed at minimum in triplicate, and the obtained data are reported as mean ± SD.

### 2.2. Characterization of Fermented Milk Samples

To obtain water-soluble extracts (WSEs), the following procedure was performed. For the samples with a pH above 4.6, the pH was adjusted to 4.6 by adding 0.75% trichloroacetic acid (TCA), and the sample was centrifuged at 10,000× *g* for 20 min at 4 °C. The supernatant was filtered through a 0.45 µm syringe filter and stored at −80 °C until further analysis.

The protein content of WSEs was determined using the Pierce BSA Protein Assay Kit (ThermoFisher, Rockford, IL, USA).

The proteolytic activity was quantified by the measurement of the amount of released amino groups in WSEs using the 2,4,6-trinitrobenzenesulfonic acid solution (TNBS, Sigma-Aldrich, St. Louis, MO, USA) method. The optical density at 340 nm was measured using a Synergy 2 microplate photometer–fluorimeter (BioTek, Winooski, VT, USA). A calibration curve was prepared using L-leucine (L-Leu) as a standard (0.1–2.0 mM). The results were expressed as L-Leu molar equivalents (mM (Leu)).

The in vitro antioxidant activity in WSEs was determined by the oxygen radical absorbance capacity fluorescence method (ORAC) using a Synergy 2 microplate photometer–fluorometer as described in Ref. [13]. The peroxyl radical was generated directly in the reaction medium during the thermal decomposition of the azo compound 2,2′-azobis (2-methylpropionamidine) dihydrochloride (AAPH, Sigma-Aldrich, St. Louis, MO, USA), initiated by incubation at 37 °C for 10 min. The antioxidant activity was expressed as the amount of Trolox (Sigma-Aldrich, St. Louis, MO, USA) molar equivalents (µM TE).

ACE-I activity in WSEs was determined by their ability to inhibit ACE (Sigma-Aldrich, St. Louis, MO, USA). o-Aminobenzoyl-Phe-Arg-Lys(dinitrophenyl)-Pro (Sigma-Aldrich, St. Louis, MO, USA) was used as a substrate with internal fluorescence quenching, as described in Ref. [13]. The 96-well, black, nonbinding polypropylene microplates (Greiner Bio One, Frickenhausen, Germany) were used. The measurements were carried out on a Synergy 2 microplate photometer–fluorometer. The half maximal inhibitory concentration (IC_50_) was expressed as mg of protein per mL.

### 2.3. Peptide Profile Analysis

The peptide profile of the WSEs was analyzed by liquid chromatography–tandem mass spectrometry (LC-MS/MS) in a system consisting of an Agilent 1100 chromatograph (Agilent Technologies, Santa Clara, CA, USA) and an LTQ-FT Ultra mass spectrometer (Thermo Scientific, Waltham, MA, USA) as described in Ref. [13]. The data are available in Supplementary Materials, Table S1.

The set analysis of peptidomics data was performed using “UpSetR” and “eulerr” R packages. The heat maps of peptidomics data were constructed using Peptigram—a web-based application for peptidomics data visualization [14]. The search for peptides with previously reported antioxidant and ACE-I activities was performed in the Milk Bioactive Peptide Database [15] and BIOPEP-UWM Database [16].

## 3. Results and Discussion

### 3.1. Growth Performance, Acidification Capability and Proteolytic Activity

**Figure 1:**
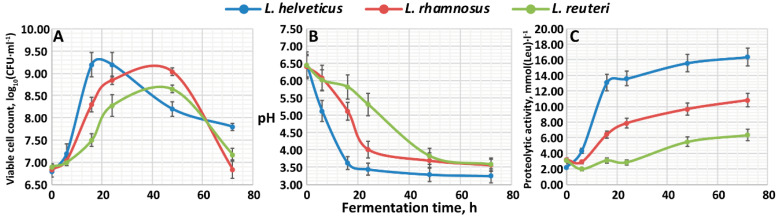
Fermentation characteristics of the *L. helveticus* NK1, *L. rhamnosus* F and *L. reuteri* LR1: (**A**)—the dynamics of change in the viable cell count; (**B**)—the dynamics of change in the pH value; (**C**)—the dynamics of change in the proteolytic activity.

To assess the growth performance during milk fermentation of *L. helveticus* NK1, *L. rhamnosus* F and *L. reuteri* LR1, milk was inoculated with corresponding LAB cultures and fermented for 72 h. The dynamics of changes in the viable cell count are shown in Figure 1A. During the fermentation process, *L. helveticus* NK1 demonstrated the highest growth rate as well as the highest maximally attainable viable cell count, (1.58 ± 0.15) × 10^9^ CFU·mL^−1^ (at 24 h), among all studied LABs. The exponential growth phase for this strain lasted until 16 h, and the stationary phase until 24 h. For the *L. rhamnosus* F, having an intermediate growth rate, the exponential growth phase lasted approximately until 24 h, and the stationary phase until 48 h; the maximally attainable viable cell count comprised (1.10 ± 0.11) × 10^9^ CFU·mL^−1^ (at 48 h). The lowest growth rate was demonstrated by *L. reuteri* LR1. This strain grew exponentially until 48 h, when it attained the maximal viable cell count of (4.50 ± 0.19) × 10^8^ CFU·mL^−1^; there was no pronounced stationary growth phase observed for this LAB (the values at 24 and 48 h were significantly different according to the Student’s *t*-test, *p* < 0.05).

The dynamics of changes in the pH values are shown in Figure 1B. As expected, the acidification capabilities of all studied LABs negatively correlated with their growth patterns, and the faster the strain grew, the greater acidification of its medium was observed. While *L. helveticus* NK1 showed a pH decrease of approximately three units in a 24 h timespan (reaching the final pH of 3.25 ± 0.20 at 72 h), *L. rhamnosus* F and *L. reuteri* LR1 decreased pH by the same amount only at the end of the fermentation. It should be noted that all performed fermentations eventually led to the coagulation of milk proteins, because of casein precipitation at a pH lower than 4.6 [17].

The degree of hydrolysis of milk proteins, which characterizes the proteolytic activity of LABs, was measured as the concentration of free amino groups expressed in L-leucine equivalents. The dynamics of changes of the proteolytic activity for the studied LABs are shown in Figure 1C. Among all studied LABs, the highest proteolytic activity was demonstrated by *L. helveticus* NK1. For this LAB, an active growth of the proteolytic activity was observed in the first 16 h of fermentation, when the proteolytic activity reached 13.07 ± 1.05 mM (Leu). Then, during the period of 16–24 h, the proteolytic activity remained constant, after which a slight increase up to 16.36 ± 1.15 mM (Leu) was observed. For the *L. rhamnosus* F and *L. reuteri* LR1, the dynamics of the proteolytic activity were different. For both of these LABs, a slight decrease in the proteolytic activity was observed in the first 6 h of fermentation—from 3.17 ± 0.22 mM (Leu) to 2.89 ± 0.19 mM (Leu) for *L. rhamnosus* F and from 3.08 ± 0.37 mM (Leu) to 1.98 ± 0.22 mM (Leu) for *L. reuteri* LR1. This can indicate that the process of the utilization of peptides, which were already present in milk, prevailed over the process of the hydrolysis of milk proteins by LAB’s proteases. The most active increase (up to 7.87 ± 0.63 and 2.86 ± 0.34 mM (Leu) for *L. rhamnosus* F and *L. reuteri* LR1, respectively) in proteolytic activity was observed from 6 to 24 h of fermentation, after which the proteolysis went slower. At the end of the fermentation, the proteolytic activities comprised 10.81 ± 0.86 mM (Leu) and 6.35 ± 0.76 mM (Leu) for the *L. rhamnosus* F and *L. reuteri* LR1, respectively.

While the strain *L. helveticus* NK1 demonstrated comparable proteolytic activity to those previously reported for this LAB species [18,19], the strain *L. rhamnosus* F studied in this work showed a higher proteolytic activity than those described in the literature—the *L. rhamnosus* PRA331 strain after 72 h of fermentation on milk had a proteolytic activity of 6.4 mM (Leu) (pH 4.0) [20]. Unfortunately, no comparable data on the proteolytic activity of *L. reuteri* have been previously published. The low proteolytic activity of *L. reuteri* LR1 can be explained by its lifestyle. Apart from the *L. helveticus* and *L. rhamnosus*, which are nomadic species, persisting for a limited time in the gut, the *L. reuteri* is strongly host-associated [21]. As such, this species does not require a potent proteolytic system on its own. Indeed, while two and one CEPs were identified in the genomes of *L. helveticus* and *L. rhamnosus* [6,22], respectively, there were no CEPs reported in the genomes of *L. reuteri* [23].

### 3.2. The Development of Antioxidant and Antihypertensive Properties

**Figure 2:**
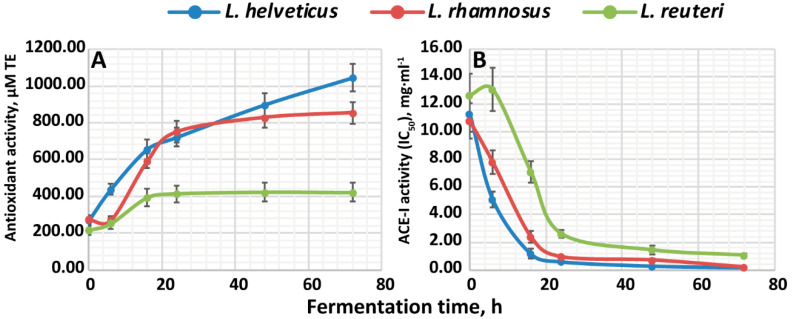
The development of antioxidant and angiotensin I-converting enzyme inhibitory (ACE-I) activities in the milk fermented by *L. helveticus* NK1, *L. rhamnosus* F and *L reuteri* LR1: (**A**)—the development of antioxidant activity; (**B**)—the development of ACE-I activity.

The development of antioxidant activity during the fermentation of milk by the studied LABs is shown in Figure 2A. The antioxidant activity of milk fermented by *L. helveticus* NK1 actively increased up to 657 ± 53 µM TE in the first 16 h of fermentation, after which it remained at the same level until 24 h. Further, a secondary increase up to 1045 ± 73 µM TE in antioxidant activity was observed. For milk fermented by *L. rhamnosus* F and *L. reuteri* LR1, the growth of antioxidant activity was not observed in the first 6 h of fermentation. From 6 to 24 h, the milk fermented by *L. rhamnosus* F demonstrated an intense increase in antioxidant activity up to 751 ± 60 µM TE, after which it continued to linearly grow until the end of fermentation, reaching 855 ± 60 µM TE. As for the milk fermented by *L. reuteri* LR1, the antioxidant activity increased up to 415 ± 46 µM TE at 24 h, after which it remained the same until the end of fermentation.

The development of ACE-I activity is shown in Figure 2B. For milk fermented by all studied LABs, the most active increase in ACE-I activity (i.e., decrease in IC_50_) was observed in the first 24 h of fermentation. At this time, for milk fermented by *L. helveticus* NK1 and *L. rhamnosus* F, the IC_50_ reached the values of 0.60 ± 0.07 and 0.95 ± 0.1 mg·mL^−1^, respectively. For milk fermented by *L. reuteri* LR1, the IC_50_ at 24 h of fermentation was 2.63 ± 0.29 mg·mL^−1^. For all studied LABs, after 24 h the IC_50_ decreased slowly and reached 0.18 ± 0.02, 0.21 ± 0.03 and 1.07 ± 0.13 mg·mL^−1^ for *L. helveticus* NK1, *L. rhamnosus* F and *L. reuteri* LR1 at 72 h of fermentation, respectively.

The observed differences between the patterns of development of the antioxidant and ACE-I activities for studied LABs can be explained by the general requirements for peptides to possess such activities. In the case of antioxidant activity, peptides should be short (up to 20 amino acids) and contain a high proportion of hydrophobic amino acids [24,25,26]. Since many peptides can satisfy these minimal requirements, any LAB can potentially produce several of them as a part of the total peptide pool. Therefore, the bigger the total pool of peptides (i.e., degree of proteolysis) in the fermented product, the greater the amount of antioxidant peptides it will contain, and the higher the antioxidant activity it will possess. In our case, the proteolytic activity decreased in the series *L. helveticus* NK1 > *L. rhamnosus* F > *L. reuteri* LR1, and the same was observed for antioxidant activity. It should be noted that the same correlation of proteolytic and antioxidant activities was previously reported in Refs. [27,28].

In contrast to antioxidant peptides, ACE-I peptides are very sequence-specific, since they have to perform competitive inhibition at the catalytic site of ACE [26]. Therefore, their production is strongly dependent on the ability of the LAB’s proteolytic system to cut at specific sequences. Consequently, only a small fraction, if any, of peptides will possess this activity independently of the degree of hydrolysis. In our study, we observed that at 24 h; the ACE-I activities of *L. helveticus* NK1 and *L. rhamnosus* F became roughly the same, despite the almost two times greater degree of proteolysis for *L. helveticus* NK1. Most probably, *L. rhamnosus* F produces more active ACE-I peptides unique to this LAB.

### 3.3. Peptide Profile

To get more insight into the process of the development of antioxidant and ACE-I activities, for each LAB, biological replicates from the 16 h of fermentation were pooled together and the peptide profile was determined using the LC-MS/MS technique. As expected, all detected peptides were mainly released from the milk’s caseins—β-, α_S1_-, α_S2_- and κ-casein; only these peptides were subjected to further analysis. The performed analysis allowed for identifying 104, 149, 98 and 128 peptides in the original milk and the milk fermented by *L. helveticus* NK1, *L. rhamnosus* F and *L. reuteri* LR1, respectively. In total, 331 unique peptides were identified in all studied samples.

**Figure 3:**
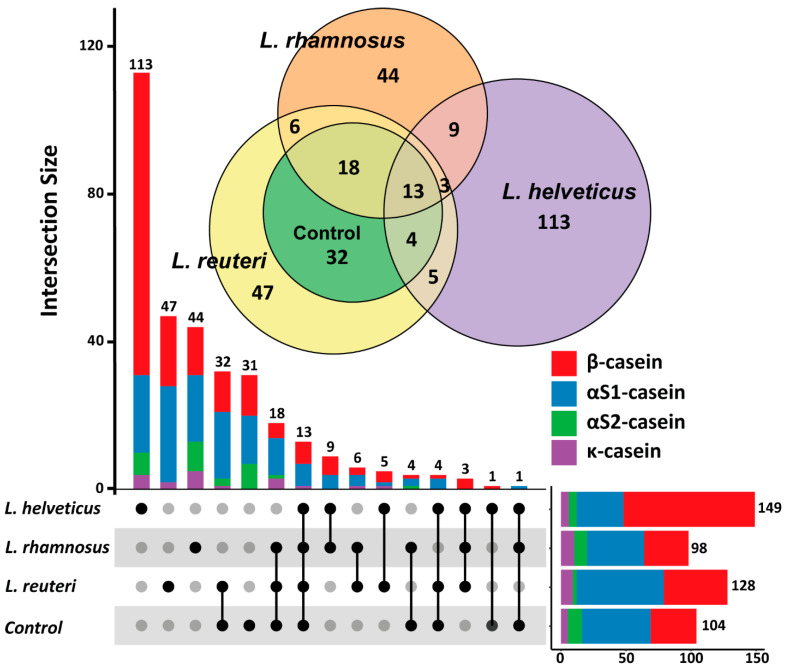
Set analysis of peptidomics data: UpSet plot, and area-proportional Euler diagrams. Unfermented milk was used as a control.

As can be seen from Figure 3, all the studied LABs significantly altered the peptide composition of the original milk. From the 104 peptides originally present in the milk, 31 peptides were totally absent in all fermentations. The greatest alteration was observed in the milk fermented by *L. helveticus* NK1. Among all peptides detected in the fermentation with this LAB, 113 were unique to it; 9 and 5 peptides were uniquely shared with *L. rhamnosus* F and *L. reuteri* LR1, respectively. For *L. rhamnosus* F, 44 peptides were unique; 9 and 6 peptides were uniquely shared with *L helveticus* NK1 and *L. reuteri* LR1, respectively. Compared to other LABs, the fermentation with *L. reuteri* LR1 contained the highest amount of the peptides originally present in the milk; however, the number of unique peptides—47—was comparable with those detected in *L. rhamnosus* F. Interestingly, *L. helveticus* NK1 mainly released unique peptides from β-casein (82 peptides), while *L. rhamnosus* F and *L. reuteri* LR1 mainly released unique peptides from α_S1_-casein (18 and 26 peptides for *L. rhamnosus* F and *L. reuteri* LR1, respectively).

**Figure 4:**
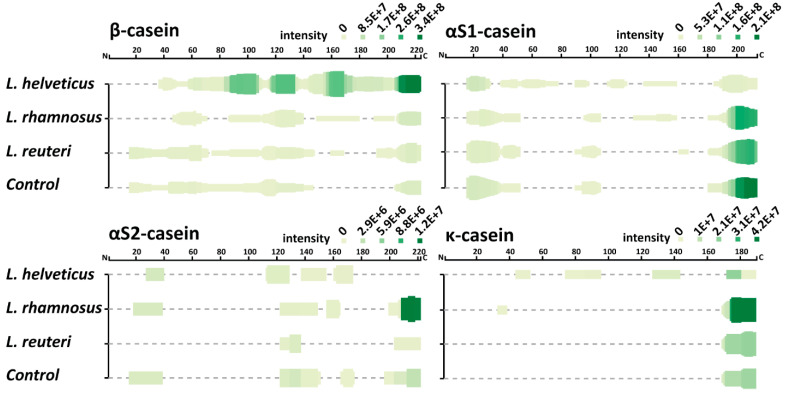
Heat map of peptidomics data. The color intensity is proportional to the normalized per protein ion intensities of peptides, with dark green indicating high peptide intensity and light green indicating low peptide intensity.

To illustrate the proteolysis patterns of the casein fractions, all detected peptides were mapped on the β-, α_S1_-, α_S2_- and κ-casein sequences (Figure 4). Generally, all studied LABs were able to cleave the β-casein along almost the entire length of its sequence; the α_S1_-casein was predominantly cleaved at its N- and C-termini, and the κ-casein at its C-terminus. In the case of α_S2_-casein, the different LABs demonstrated the most different patterns of cleavage, which is especially apparent at the protein’s termini: *L. helveticus* NK1 did not produce peptides from both termini of the protein; *L. rhamnosus* F was able to cleave both N- and C-termini; and *L. reuteri* LR1 performed cleavages only at the C-terminus.

As a result of the search for the determined peptides in publicly available databases and published literature, 22 complete matches were found (Table 1). While 14 peptides with proven ACE-I activity were determined in the milk fermented by *L. helveticus* NK1, for *L. rhamnosus* F and *L. reuteri* LR1 this number was only 4 and 10 peptides, respectively. Although, the smaller number of ACE-I peptides in *L. reuteri* LR1 well explained its smaller ACE-I activity, for *L. rhamnosus* F, having an ACE-I comparable to *L. helveticus* NK1, the data again suggested the presence of unique, previously unreported peptides.

To identify potential ACE-I peptides from *L. rhamnosus* F, its unique peptides were searched for specific ACE-I patterns. Previously, it was shown that peptides that possess in their sequence an aromatic residue at the C-terminal position are better ACE inhibitors [36]. Moreover, the location of aliphatic, basic and aromatic residues in the penultimate position, and proline, aromatic and aliphatic residues in the last position, was proposed as the strong ACE-I pattern [37]. In our case, five peptides satisfying these requirements were identified (Table 2). Interestingly, all these peptides originated from the C-terminus of α_S2_-casein.

## 4. Conclusions

In the current work, different abilities of LABs to develop antioxidant and ACE-inhibitory properties during milk fermentation were observed. For all studied LABs, the development of antioxidant activity was correlated with the degree of milk proteolysis. The strain demonstrating the highest proteolytic as well as antioxidant activities was *L. helveticus* NK1, followed by *L. rhamnosus* F and *L. reuteri* LR1. Although the proteolytic activity of *L. helveticus* NK1 was two times higher than that for *L. rhamnosus* F, the milk fermented by these strains showed comparable ACE inhibition after 24 h of fermentation. The analysis of the peptide profile of the obtained products of fermentation allowed us to hypothesize that some previously unreported peptides should be produced by *L. rhamnosus* F. The detailed analysis of the *L. rhamnosus* F peptide profile allowed us to suggest that, most probably, its ACE-inhibitory peptides originated from the C-terminus of the α_S2_-casein. These peptides are promising candidates for the further testing of their antihypertensive properties in vivo using an appropriate pure line of rats (e.g., spontaneously hypertensive rats, SHR).

## Figures and Tables

**Table 1:** Bioactive peptides identified in milk samples fermented by *Lactobacillus* spp. strains after 16 h of milk fermentation *.

Peptide	Protein Fragment	MS Extracted Ion Intensity				Activity	Ref.
		Control	Lr LR1	Lrh F	Lhel NK1		
DKIHPF	β-CN f (47–52)	nd	nd	1.34 × 10^6^	3.33 × 10^6^	ACE-I/Antioxidant	[29]
VVPPFLQPE	β-CN f (83–91)	nd	nd	nd	1.44 × 10^6^	ACE-I	[30]
YPFPGPIPN	β-CN f (60–68)	nd	2.77 × 10^5^	nd	nd	ACE-I	[31]
NIPPLTQTPV	β-CN f (73–82)	nd	nd	nd	6.56 × 10^6^	ACE-I	[29]
TQTPVVVPPFLQPE	β-CN f (78–91)	nd	nd	nd	1.25 × 10^6^	Antioxidant	[32]
DVENLHLPLPLLQSWM	β-CN f (129–144)	nd	nd	nd	8.21 × 10^5^	ACE-I	[33]
LHLPLPLLQSW	β-CN f (133–143)	nd	nd	nd	1.29 × 10^5^	ACE-I	[29,30]
SLSQSKVLPVPQK	β-CN f (164–176)	nd	nd	nd	9.27 × 10^6^	Antioxidant	[32]
KVLPVPQ	β-CN f (169–175)	nd	nd	nd	2.17 × 10^5^	ACE-I	[34]
LLYQEPVLGPVRGPFPIIV	β-CN f (191–209)	8.89 × 10^6^	8.52 × 10^5^	nd	1.28 × 10^8^	ACE-I/Antioxidant	[32]
YQEPVLGPVRGPFP	β-CN f (193–206)	nd	3.42 × 10^6^	1.73 × 10^5^	nd	ACE-I	[32]
QEPVLGPVRGPFPIIV	β-CN f (194–209)	nd	1.8 × 10^6^	3.04 × 10^7^	1.13 × 10^8^	ACE-I/Antioxidant	[32]
EPVLGPVRGPFP	β-CN f (195–206)	nd	4.17 × 10^5^	nd	nd	ACE-I	[35]
GPVRGPFPIIV	β-CN f (199–209)	1.17 × 10^6^	7.49 × 10^6^	2.66 × 10^4^	2.42 × 10^7^	ACE-I	[30]
RPKHPIKHQ	α_S1_-CN f (1–9)	nd	4.09 × 10^4^	nd	6.06 × 10^6^	ACE-I	[31]
EVLNENLLRF	α_S1_-CN f (14–23)	nd	1.46 × 10^5^	nd	nd	ACE-I	[32]
FVAPFPEVFGKE	α_S1_-CN f (24–35)	nd	nd	nd	3.72 × 10^5^	ACE-I/Antioxidant	[30]
VAPFPEVFGKE	α_S1_-CN f (25–35)	nd	nd	nd	4.45 × 10^5^	ACE-I	[32]
LYQGPIVLNPWDQVK	α_S2_-CN f (99–113)	nd	nd	nd	3.9 × 10^5^	ACE-I	[32]
NAVPITPT	α_S2_-CN f (115–122)	6.11 × 10^5^	5.81 × 10^5^	nd	nd	ACE-I	[32]
KYIPIQYVL	κ-CN f (30–38)	nd	nd	nd	4.47 × 10^5^	Antioxidant	[33]
VQVTSTAV	κ-CN f (162–169)	3.21 × 10^5^	3.15 × 10^5^	nd	nd	ACE-I	[30]

* Unfermented milk was used as a control. Abbreviations: β-CN—β-casein; α_S1_-CN—αS1-casein; α_S2_-CN—α_S2_-casein; κ-CN—κ-casein. Lr LR1—*L. reuteri* LR1; Lrh F—*L. rhamnosus* F; Lhel NK1—*L. helveticus* NK1.

**Table 2:** The α_S2_-casein (α_S2_-CN) peptides identified in milk samples fermented by *L. rhamnosus* F strains after 16 h of milk fermentation.

Peptide *	Protein Fragment	MS Extracted Ion Intensity
QHQKAMKP**W**	α_S2_-CN f (200–208)	1.88 × 10^4^
PWIQPKTKVIPYVR**YL**	α_S2_-CN f (207–222)	1.66 × 10^5^
IQPKTKVIPYVR**YL**	α_S2_-CN f (209–222)	1.14 × 10^7^
IQPKTKVIP**Y**	α_S2_-CN f (209–218)	1.44 × 10^5^
KVIPYVR**YL**	α_S2_-CN f (214–222)	7.53 × 10^4^

* Typical for ACE-I peptides amino acid residues are shown in bold.

## Data Availability

Data is contained within the article or supplementary material.

## Supplementary Materials

The following are available online at https://www.mdpi.com/2304-8158/10/1/17/s1, Table S1: Results of the peptidomics analysis.

## References

[1] Nasri H., Baradaran A., Shirzad H., Rafieian-Kopaei M. (2014). New Concepts in Nutraceuticals as Alternative for Pharmaceuticals. Int. J. Prev. Med..

[2] Mine Y., Li-Chan E.C., Jiang B. (2010). Biologically Active Food Proteins and Peptides in Health: An Overview. Bioactive Proteins and Peptides as Functional Foods and Nutraceuticals.

[3] Chanda S., Tiwari R.K., Kumar A., Singh K. (2019). Nutraceuticals Inspiring the Current Therapy for Lifestyle Diseases. Adv. Pharmacol. Sci..

[4] Beltrán-Barrientos L., Hernández-Mendoza A., Torres-Llanez M., González-Córdova A., Vallejo-Cordoba B. (2016). Invited review: Fermented milk as antihypertensive functional food. J. Dairy Sci..

[5] Séverin S., Wenshui X. (2005). Milk Biologically Active Components as Nutraceuticals: Review. Crit. Rev. Food Sci. Nutr..

[6] Brown L., Pingitore E.V., Mozzi F., Saavedra L., Villegas J.M., Hebert E. (2017). Lactic Acid Bacteria as Cell Factories for the Generation of Bioactive Peptides. Protein Pept. Lett..

[7] Sánchez A., Vázquez A. (2017). Bioactive peptides: A review. Food Qual. Saf..

[8] Raveschot C., Cudennec B., Coutte F., Flahaut C., Fremont M., Drider D., Dhulster P. (2018). Production of Bioactive Peptides by *Lactobacillus* Species: From Gene to Application. Front. Microbiol..

[9] Tagliazucchi D., Martini S., Solieri L. (2019). Bioprospecting for Bioactive Peptide Production by Lactic Acid Bacteria Isolated from Fermented Dairy Food. Fermentation.

[10] Liong M.-T. (2015). Beneficial Microorganisms in Food and Nutraceuticals.

[11] Fedorova T.V., Vasina D.V., Begunova A.V., Rozhkova I.V., Raskoshnaya T.A., Gabrielyan N.I. (2018). Antagonistic Activity of Lactic Acid Bacteria *Lactobacillus* spp. against Clinical Isolates of *Klebsiella pneumoniae*. Appl. Biochem. Microbiol..

[12] Begunova A.V., Rozhkova I.V., Zvereva E.A., Glazunova O.A., Fedorova T.V. (2019). Lactic and Propionic Acid Bacteria: The Formation of a Community for the Production of Functional Products with Bifidogenic and Hypotensitive Properties. Appl. Biochem. Microbiol..

[13] Torkova A.A., Ryazantseva K.A., Agarkova E.Y., Kruchinin A.G., Tsentalovich M.Y., Fedorova T.V. (2017). Rational design of enzyme compositions for the production of functional hydrolysates of cow milk whey proteins. Appl. Biochem. Microbiol..

[14] Manguy J., Jehl P., Dillon E.T., Davey N.E., Shields D.C., Holton T.A. (2017). Peptigram: A web-based application for peptidomics data visualization. J. Proteome Res..

[15] Nielsen S.D., Beverly R.L., Qu Y., Dallas D.C. (2017). Milk bioactive peptide database: A comprehensive data-base of milk protein-derived bioactive peptides and novel visualization. Food Chem..

[16] Minkiewicz P., Iwaniak A., Darewicz M. (2019). BIOPEP-UWM database of bioactive peptides: Current opportunities. Int. J. Mol. Sci..

[17] Raak N., Rohm H., Jaros D. (2017). Enzymatic Cross-Linking of Casein Facilitates Gel Structure Weakening Induced by Overacidification. Food Biophys..

[18] Giacometti Cavalheiro F., Parra Baptista D., Domingues Galli B., Negrão F., Nogueira Eberlin M., Lúcia Gigante M. (2020). High protein yogurt with addition of *Lactobacillus helveticus*: Peptide profile and angiotensin-converting enzyme ACE-inhibitory activity. Food Chem..

[19] Pihlanto A., Virtanen T., Korhonen H.J.T. (2010). Angiotensin I converting enzyme (ACE) inhibitory activity and antihypertensive effect of fermented milk. Int. Dairy J..

[20] Solieri L., De Vero L., Tagliazucchi D. (2018). Peptidomic study of casein proteolysis in bovine milk by *Lactobacillus casei* PRA205 and *Lactobacillus rhamnosus* PRA331. Int. Dairy J..

[21] Duar R.M., Lin X.B., Zheng J., Martino M.E., Grenier T., Pérez-Muñoz M.E., Leulier F., Gänzle M., Walter J. (2017). Lifestyles in transition: Evolution and natural history of the genus *Lactobacillus*. FEMS Microbiol. Rev..

[22] Sadat-Mekmene L., Genay M., Atlan D., Lortal S., Gagnaire V. (2011). Original features of cell-envelope pro-teinases of *Lactobacillus helveticus*. A review. Int. J. Food Microbiol..

[23] Liu M., Bayjanov J.R., Renckens B., Nauta A., Siezen R.J. (2010). The proteolytic system of lactic acid bacteria revisited: A genomic comparison. BMC Genom..

[24] Sarmadi B.H., Ismail A. (2010). Antioxidative peptides from food proteins: A review. Peptides.

[25] Pihlanto A. (2006). Antioxidative peptides derived from milk proteins. Int. Dairy J..

[26] Udenigwe C.C., E Aluko R. (2011). Food Protein-Derived Bioactive Peptides: Production, Processing, and Potential Health Benefits. J. Food Sci..

[27] Ramesh V., Kumar R., Singh R.R.B., Kaushik J.K., Mann B. (2011). Comparative evaluation of selected strains of lactobacilli for the development of antioxidant activity in milk. Dairy Sci. Technol..

[28] Virtanen T., Pihlanto A., Akkanen S., Korhonen H.J. (2007). Development of antioxidant activity in milk whey during fermentation with lactic acid bacteria. J. Appl. Microbiol..

[29] Gobbetti M., Ferranti P., Smacchi E., Goffredi F., Addeo F. (2000). Production of angiotensin-I-converting-enzyme-inhibitory peptides in fermented milks started by *Lactobacillus delbrueckii* subsp. bulgaricus SS1 and *Lactococcus lactis* subsp. cremoris FT4. Appl. Environ. Microbiol..

[30] Contreras M.D.M., Carrón R., Montero M.J., Ramos M., Recio I. (2009). Novel casein-derived peptides with antihypertensive activity. Int. Dairy J..

[31] Saito T., Nakamura T., Kitazawa H., Kawai Y., Itoh T. (2000). Isolation and structural analysis of antihypertensive peptides that exist naturally in gouda cheese. J. Dairy Sci..

[32] Ali E., Nielsen S.D., Aal S.A.-E., El-Leboudy A., Saleh E., Lapointe G. (2019). Use of Mass Spectrometry to Profile Peptides in Whey Protein Isolate Medium Fermented by *Lactobacillus helveticus* LH-2 and *Lactobacillus acidophilus* La-5. Front. Nutr..

[33] Abdel-Hamid M., Romeih E., Gamba R.R., Nagai E., Suzuki T., Koyanagi T., Enomoto T. (2019). The biological activity of fermented milk produced by *Lactobacillus casei* ATCC 393 during cold storage. Int. Dairy J..

[34] Maeno M., Yamamoto N., Takano T. (1996). Identification of an antihypertensive peptide from casein hydrolysate produced by a proteinase from *Lactobacillus helveticus* CP790. J. Dairy Sci..

[35] Hayes M., Stanton C., Slattery H., O’Sullivan O., Hill C., Fitzgerald G.F., Ross R.P. (2007). Casein Fermentate of *Lactobacillus animalis* DPC6134 Contains a Range of Novel Propeptide Angiotensin-Converting Enzyme Inhibitors. Appl. Environ. Microbiol..

[36] Ong L., Shah N. (2008). Release and identification of angiotensin-converting enzyme-inhibitory peptides as influenced by ripening temperatures and probiotic adjuncts in Cheddar cheeses. LWT.

[37] Pihlanto-Leppälä A., Koskinen P., Piilola K., Tupasela T., Korhonen H. (2000). Angiotensin I-converting enzyme inhibitory properties of whey protein digests: Concentration and characterization of active peptides. J. Dairy Res..

